# Inhibiting miR–618 Promotes Keratinocytes Proliferation and Migration to Enhance Wound Healing in Mice

**DOI:** 10.3390/ijms25147617

**Published:** 2024-07-11

**Authors:** Lingling Wu, Wenjun Fu, Yiyang Cao, Shuo Zhao, Yuchen Zhang, Xiaonan Li, Naijun Dong, Wenxin Qi, Rabia Malik, Jiao Wang, Robert Chunhua Zhao

**Affiliations:** 1 School of Life Sciences, Shanghai University, Shanghai 200444, China; 19990510wll@shu.edu.cn (L.W.); reddrop@shu.edu.cn (W.F.); 15239050331@shu.edu.cn (Y.C.); zhaoshuo@shu.edu.cn (S.Z.); 21724853@shu.edu.cn (Y.Z.); rywqwxwx@163.com (X.L.); dongnaijun@shu.edu.cn (N.D.); qiwenxin@shu.edu.cn (W.Q.); rabiamalik78112@gmail.com (R.M.); 2 School of Medicine, Shanghai University, Shanghai 200444, China; 3 Institute of Basic Medical, Chinese Academy of Medical Sciences, School of Basic Medicine, Peking Union Medical College, Beijing 100005, China; 4 Centre of Excellence in Tissue Engineering, Chinese Academy of Medical Sciences, Beijing 100005, China; 5 Beijing Key Laboratory of New Drug Development and Clinical Trial of Stem Cell Therapy (BZ0381), Beijing 100005, China

**Keywords:** wound healing, miR–618, epithelial–mesenchymal transition

## Abstract

The delay in wound healing caused by chronic wounds or pathological scars is a pressing issue in clinical practice, imposing significant economic and psychological burdens on patients. In particular, with the aging of the population and the increasing incidence of diseases such as diabetes, impaired wound healing is one of the growing health problems. MicroRNA (miRNA) plays a crucial role in wound healing and regulates various biological processes. Our results show that miR–618 was significantly upregulated during the inflammatory phase of wound healing.Subsequently, miR–618 promotes the secretion of pro–inflammatory cytokines and regulates the proliferation and migration of keratinocytes. Mechanistically, miR–618 binds to the target gene–*Atp11b* and inhibits the PI3K–Akt signaling pathway, inhibiting the epithelial–mesenchymal transition (EMT) of keratinocytes. In addition, the PI3K–Akt signaling pathway induces the enrichment of nuclear miR–618, and miR–618 binds to the promoter of *Lin7a* to regulate gene transcription. Intradermal injection of miR–618 antagomir around full–thickness wounds in peridermal mice effectively accelerates wound closure compared to control. In conclusion, miR–618 antagomir can be a potential therapeutic agent for wound healing.

## 1. Introduction

Skin has a vital barrier protection function which maintains homeostasis. Demographically, the number of patients with chronic wounds and healing disorders is on the rise, placing a more significant burden on human health and the economy [1]. Acute wounds (such as burn wounds and surgical wounds) [2] and chronic skin wounds (such as pressure ulcers and diabetic foot ulcers) [3,4,5] are urgent problems for clinical dermatology.

EMT events occur during injury–repair–related processes in which epithelial cells differentiate into new fibroblast–like cells to rebuild tissue after trauma and inflammatory damage [4,6]. These cells exhibit epithelial–specific morphological and molecular markers, such as E–cadherin, but also express mesenchymal markers: N–cadherin, α–smooth muscle actin (α–SMA), and Vimentin [6,7]. EMT activates and mobilizes keratinocytes proliferation and migration, enhancing cell motility and bone rearrangement to promote skin wound healing [8].

Tissue–specific miRNAs are often associated with disease–specific tissues. miRNAs are involved in the regulation of many biological processes, including differentiation, proliferation, apoptosis, and cell migration [9]. The skin injury repair process includes the hemostasis, inflammatory, proliferative, and remodeling phase [4]. Multiple biological pathways are aimed at restoring tissues and maintaining skin integrity. Many studies have also revealed the role of different miRNAs in different skin cell lines and wound healing [10]. Most studies about miR–618 were related to its mechanism, function, and therapeutic efficacy in cancer therapy [11,12,13,14,15]. In addition, miR–618 has also been found to be associated with skin fibrosis [16].

In this study, we first analyzed the differential expression of miRNAs in wound tissue during the inflammatory phase. The result showed that miR–618 was dynamically upregulated in the inflammatory phase. Therefore, we focused on the role of miR–618 in skin wound healing. We found that miR–618 inhibits keratinocytes proliferation and migration by inhibiting downstream target gene–*Atp11b*, and regulates keratinocytes’ EMT through the PI3K–Akt signaling pathway. We also found that the PI3K–Akt signaling pathway induces miR–618 nuclear enrichment, and miR–618 binds to the *Lin7a* promoter region. Our findings suggest that miR–618 plays a crucial role in skin wound healing by inhibiting keratinocytes proliferation and migration.

## 2. Results

### 2.1. Mapping of miRNA Expression in the Inflammatory Phase of Human Skin Wounds

**Figure 1:**
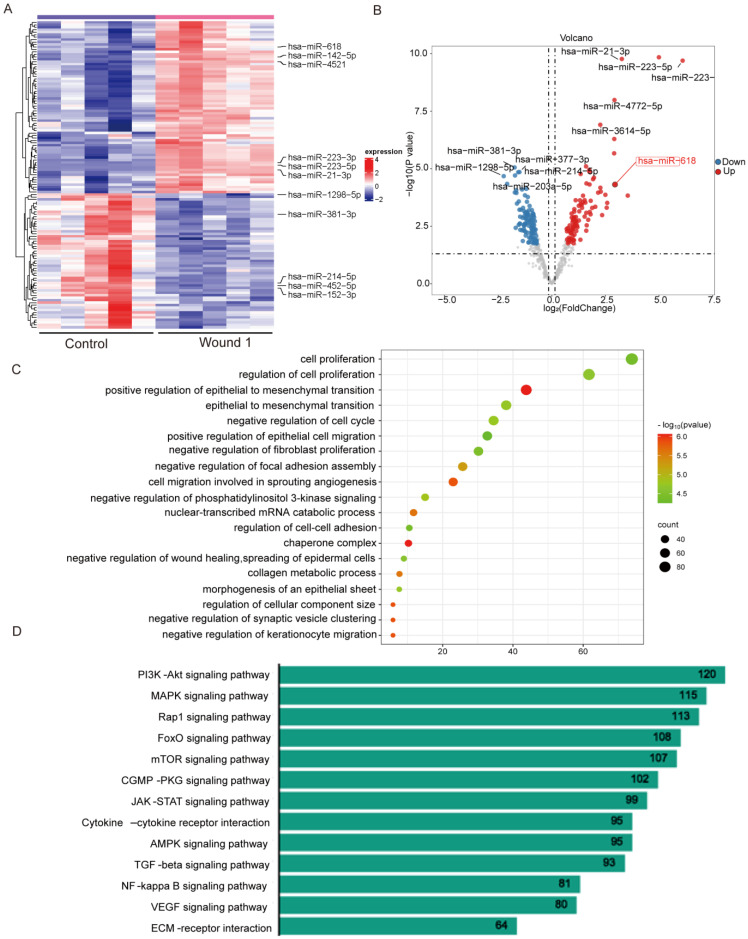
Mapping of miRNA expression in the inflammatory phase of human skin wounds. (**A**) Heatmap showing differentially expressed miRNA expression in the inflammatory phase of human skin wound healing. (**B**) Volcanic map of miRNA expression between normal skin tissue and wound tissue in the inflammatory phase. (**C**,**D**) GO and KEGG functional enrichment analysis of different expression of miRNAs. (*n* = 5).

miRNAs are dysregulated during wound healing in human skin and are cell–specific [17]. In order to better understand the potential connection between miRNAs and the healing of human skin wounds, we explored a comprehensive gene expression (GEO) database comparing the expression of 562 miRNAs in normal skin tissue and inflammatory stage wound tissue from five healthy donors (Figure 1A). Compared with normal skin tissue, there were 126 differential miRNAs in wound tissue during the inflammatory phase, of which, 55 miRNAs were significantly downregulated and 71 miRNAs were significantly upregulated (|log_2_FC| > 0.25, *p*-value < 0.05) (Figure 1B). Notably, several miRNAs identified have previously been shown to modulate various biological processes associated with skin wound healing (Table 1). miR–21–3p, miR–223, miR-4521, miR-618 and miR–92-3p were upregulated and miR–381–3p, miR–452–5p, and miR–214–5p were downregulated. In addition, we also analyzed miRNA changes during the transition from the inflammatory to the proliferative phase. There were 235 different miRNAs in the transition from the inflammatory stage to proliferative stage, of which, 115 miRNAs were significantly upregulated and 120 miRNAs were significantly downregulated (Figure S1A,B). To identify the mechanisms which these differential miRNAs in wound healing, we investigated miEAA (https://ccb-compute2.cs.uni-saarland.de/mieaa2/, accessed on 10 May 2023) and carried out the functional enrichment analysis of differential miRNA, which was mainly enriched by cell migration, negative regulation of cell growth, and response to wounding (Figure 1C). These functional enrichment results suggest that differential miRNAs may regulate wound healing by regulating the motility of skin–related cells, such as cell proliferation and migration. Through Gene Ontology (GO) functional enrichment analysis, we found that differential miRNAs mainly affect skin cell proliferation and the migratory ability to participate in wound healing. To further explore the specific biological mechanisms of differential miRNAs, we used Kyoto Encyclopedia of Genes and Genomes (KEGG) to perform functional enrichment analysis of the signaling pathways in which differential miRNAs may be involved. The results showed that most of the genes were enriched in the PI3K–Akt signaling pathway, MAPK signaling pathway, Rap1 signaling pathway, FoxO signaling pathway, and mTOR signaling pathway (Figure 1D). miRNA is widely expressed in the cytoplasm and nucleus and regulates gene expression at transcriptional and post–transcriptional levels. Therefore, we speculate that differential miRNAs may regulate the function of skin–related cells by participating in the transmission of cell signaling pathways, affecting the repair ability of keratinocytes during wound healing.

### 2.2. miR–618 Regulates the Inflammatory and Proliferative Phases of Human Skin Wound

**Figure 2:**
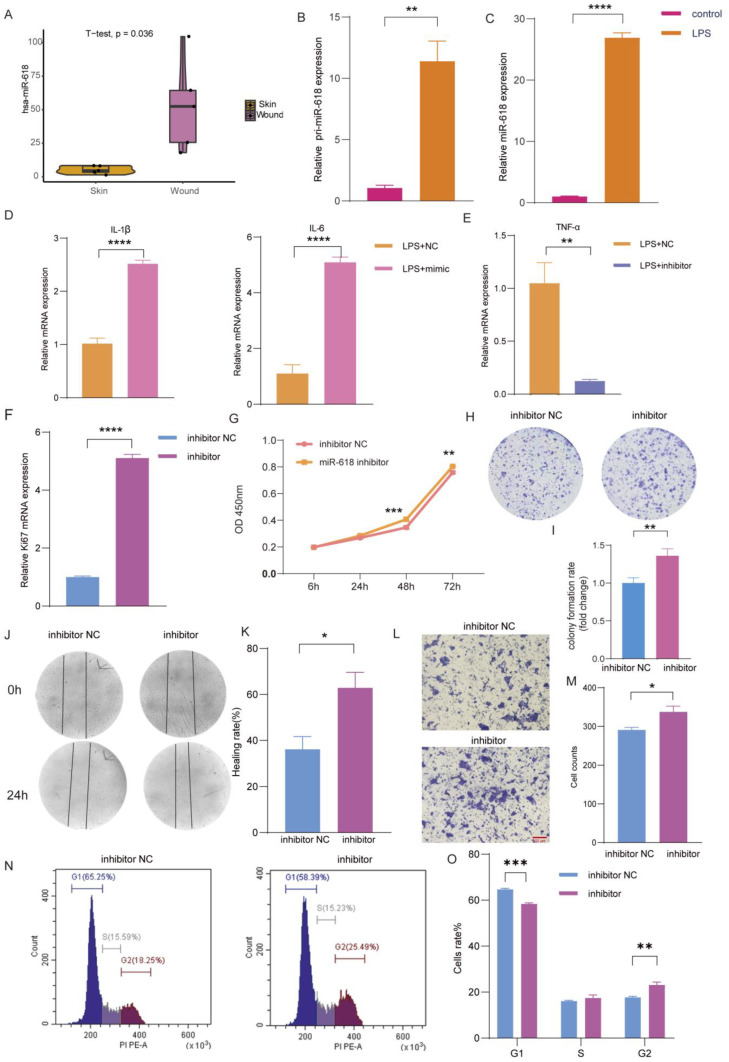
miR–618 regulates the inflammatory and proliferative phases of human skin wound. (**A**) The expression level of miR–618 was obtained from the GSE174661 database in normal tissue and wound inflammatory tissue (*n* = 5). (**B**,**C**) mRNA expression levels of pri–miR–618 and miR–618 in LPS–treated keratinocytes. (**D**) mRNA expression levels of pro–inflammatory factors *IL–1β* and *IL–6* in keratinocytes treated with LPS and transfected with miR–618 mimic and NC. (**E**) mRNA expression level of pro–inflammatory factor (*TNF–α*) in keratinocytes which were treated with LPS and transfected with miR–618 inhibitor and NC. (**F**) mRNA expression level of *Ki67* in keratinocyte transfected with miR–618 mimic and NC. (**G**) CCK8 assay was performed to evaluate the proliferation ability of transfected keratinocytes. (**H**,**I**) Representative images of colony formation assay of transfected keratinocytes. Statistical analysis of colony numbers was performed using Image J 1.53. (**J**,**K**) Scratch assay was performed to detect the migration ability of transfected keratinocytes. Images were taken at 0 h and 24 h after scratching. Statistical analysis of scratch healing was performed using Image J 1.53. (**L**,**M**) Transwell assay to determine the migration ability of transfected keratinocytes. Scale: 100 µm. The number of Transwell migration cells was analyzed using Image J 1.53. (**N**,**O**) Flow cytometry was performed to analyze the cell cycle of transfected keratinocytes. Data are expressed as the mean ± SD. * *p* < 0.05, ** *p* < 0.01, *** *p* < 0.001, **** *p* < 0.0001. *n* = 3.

Among these notably divergent miRNAs, the expression level of miR-618 has been reported to exhibit a marked elevation in blood samples procured from patients sustaining severe injuries [18] and miR–618 has been reported to be associated with skin fibrotic lesions [16]. This finding underscores the potential significance of miR-618 in the context of acute trauma and its potential as a biomarker for assessing injury severity. However, its mechanism of action in skin injury repair remains unclear. Since the expression of miR–618 was significantly upregulated during the inflammatory phase of wound healing (Figure 2A). A lipopolysaccharide (LPS)–induced inflammation cell model was constructed to further study the role of miR–618 in the inflammatory healing phase of wound healing. The expression levels of inflammatory cytokines *IL–1β* and *TNF–α* were significantly increased after LPS induction for 24 h (Figure S2A). Therefore, the inflammatory cell model was subsequently constructed by using LPS–induced cells for 24 h. Next, qRT–PCR results showed that the inflammatory factor storm significantly increased the expression levels of pri–miR–618 and miR–618 (Figure 2B,C). These data suggest that the inflammatory response may modulate the biogenesis of miR–618 at the transcriptional level and the processing of the primary transcript. In addition, miR–618 mimic was simultaneously transfected in the LPS–induced inflammatory cell model. The qRT–PCR results showed that the expression levels of pro–inflammatory factors *IL–1β* and *IL–6* were significantly increased in the LPS + mimic group compared with the LPS group (Figure 2D). Conversely, inhibiting the expression of miR–618 reduced the expression of *TNF–α* in inflammatory cell and reduced the inflammatory response (Figure 2E). These results suggest that the overexpression of miR–618 stimulates the expression of inflammatory cytokines and that miR–618 is involved in the inflammatory phase of wound healing. We overexpressed or inhibited the expression of miR–618 in keratinocytes to explore the effect on proliferation. Initially, the proliferation marker *Ki–67* was upregulated when miR–618 expression was inhibited (Figure 2F) but downregulated when miR–618 was overexpressed (Figure S2B). Furthermore, the result of cell counting Kit–8 (CCK8) showed that decreasing the expression of miR–618 promoted keratinocyte proliferation (Figure 2G), while increasing the expression of miR–618 produced the opposite result (Figure S2C). Colony formation assay also showed that decreasing the expression of miR–618 promoted the proliferation of keratinocytes, while increasing the expression of miR–618 produced the opposite result (Figure 2H,I and Figure S2D,E). Decreasing the expression of miR–618 significantly promoted scratch healing (Figure 2J,K), while miR–618 overexpression significantly inhibited wound healing (Figure S2F,G). Meanwhile, the Transwell migration assay also showed that decreasing the expression of miR–618 promoted keratinocyte migration (Figure 2L,M), while miR–618 overexpression decreased keratinocyte migration (Figure S2H,I). Finally, cell cycle experiments showed that miR–618 regulates cell cycle progression, thereby regulating cell motor function (Figure 2N,O and Figure S2J,K). The above data indicated that miR–618 is a negative regulator of wound healing and is involved in wound inflammation and proliferation repair.

### 2.3. Atp11b as a Target Gene of miR–618 

**Figure 3:**
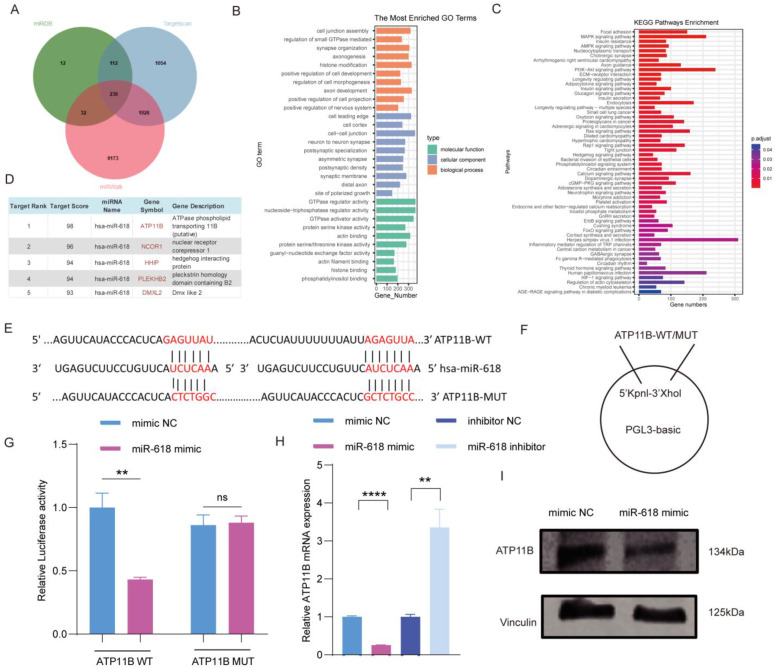
*Atp11b* as a target gene of miR–618. (**A**) Venn diagram showed overlapping miR–618 target genes in miRWALK, miRDB, and TargetScan7 databases. (**B**) GO functional enrichment analysis of miR–618 target genes, including cellular components, biological processes, and molecular functions. (**C**) KEGG enrichment analysis of miR–618 target genes. Colors represent *p*–values, with darker red representing more significance. (**D**) miRDB predicted the target score of *Atp11b* in the miR–618 target genes. (**E**–**G**) Luciferase reporter assay of HEK–293T cells transfected with ATP11B–WT or MUT and miR–618 mimic or NC. Relative luciferase activities were normalized against the Renilla luciferase activities. (**H**) mRNA expression level of *Atp11b* in keratinocytes transfected with miR–618 mimic, inhibitor, and NC. (**I**) Protein expression level of ATP11B in keratinocytes transfected with miR–618 mimic and NC. Data are expressed as the mean ± SD. ^ns^ *p* > 0.05, ** *p* < 0.01, **** *p* < 0.0001. *n* = 3.

miRNA binds to the 3′–non–coding region (3′–UTR) of the target gene, inhibiting the translation of the target gene. Targetscan7 (http://www.targetscan.org/vert_70/, accessed on 10 May 2023), miRWalk (http://zmf.umm.uni-heidelberg.de/apps/zmf/mirwalk2/, accessed on 10 May 2023), and miRDB (http://mirdb.org/, accessed on 10 May 2023) were used to predict 236 potential target genes for miR–618 (Figure 3A). GO functional enrichment was performed to analyze target genes of miR–618, and the results showed that the target genes functions were mainly enriched in cell junction assembly, histone modification, axonal development, cell leading edge, and cell–cell junction (Figure 3B). KEGG analysis showed that the functions of differential genes were mainly enriched in focal adhesion, the MAPK signaling pathway, and the PI3K–Akt signaling pathway (Figure 3C). These findings suggest that miR–618 may impact skin–related cell function through the regulation of cell signaling pathways. We screened these target genes and found that *Atp11b*, one of the P4–ATPase families, has the highest targeting score (Figure 3D), and it has been reported that P4–ATPase is involved in cellular inflammatory response [19,20,21]. Initially, to determine whether *Atp11b* is a direct target gene of miR–618, we cloned the specific binding sites and mutation sites of ATP11B to construct Luc–ATP11B–wild type (WT) and Luc–ATP11B–mutation type (MUT) (Figure 3E,F). The result of the double luciferase reporter gene assay showed that after transfection with miR–618 mimic, luciferase activity decreased in the Luc–ATP11B–WT group and there was no change in the Luc–ATP11B–MUT group (Figure 3G). This indicates that miR–618 interacts with ATP11B. In addition, the mRNA expression level of *Atp11b* decreased after transfection with miR–618 mimic, but increased after transfection with miR–618 inhibitor (Figure 3H). In addition, the overexpression of miR–618 significantly reduced the protein expression level of ATP11B (Figure 3I). Collectively, these findings suggest that *Atp11b* as a target gene of miR–618.

### 2.4. Silencing ATP11B Inhibits Keratinocytes Proliferation and Migration

**Figure 4:**
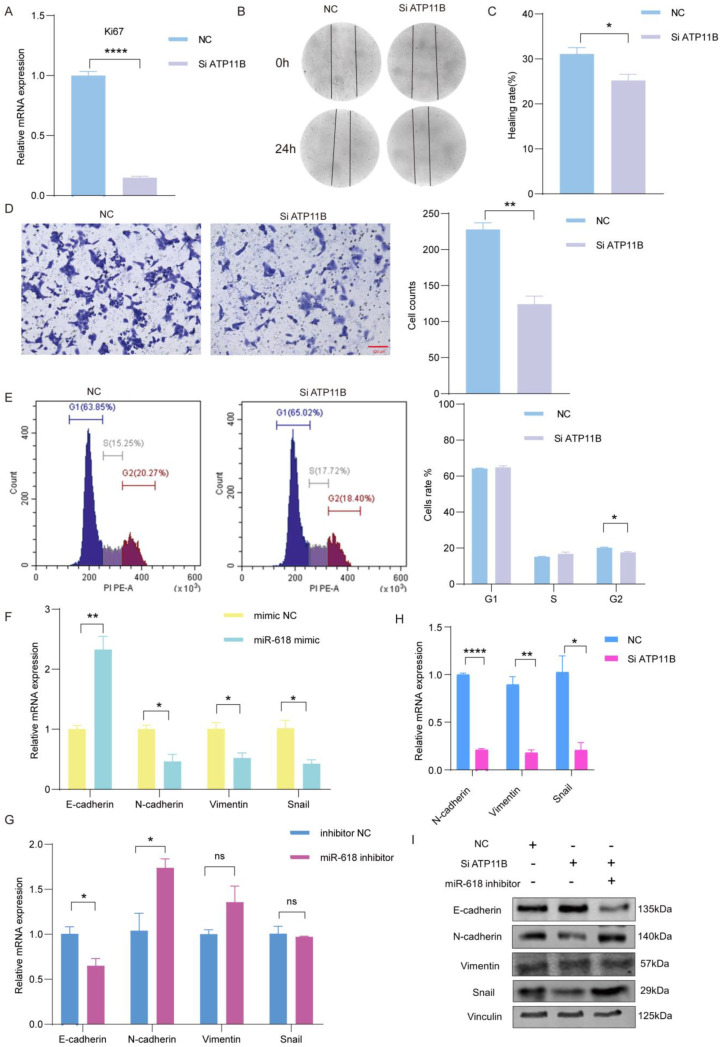
Silencing ATP11B inhibits keratinocytes proliferation and migration. (**A**) mRNA expression level of *Ki67* in keratinocytes transfected with Si ATP11B and NC. (**B**) Scratch assay was performed to detect the migration ability of transfected keratinocytes. Images were taken at 0 h and 24 h after scratching. (**C**) Quantitation of scratch healing assay at 24 h. (**D**) Transwell assay analysis of the migration ability of transfected keratinocytes. Scale: 100 µm. The number of Transwell migration cells was analyzed using Image J 1.53. (**E**) Flow cytometry was performed to analyze the cell cycle of transfected keratinocytes. The percentage of cells in the G1, S, and G2 phases of the cell cycle was displayed. (**F**) qPCR assay was used to detect the mRNA expression levels of EMT−related genes in keratinocytes after the overexpression of miR−618. (**G**) qPCR assay was used to detect the mRNA expression levels of EMT−related genes in keratinocytes after the inhibition of miR−618. (**H**) qPCR assay was used to detect the mRNA expression levels of EMT−related genes in keratinocytes after silencing ATP11B. (**I**) Protein expression levels of EMT−related genes in keratinocytes after co–transfection. Data are expressed as the mean ± SD. ^ns^ *p* > 0.05, * *p* < 0.05, ** *p* < 0.01, **** *p* < 0.0001. *n* = 3.

To determine whether the target gene *Atp11b* affects the proliferation and migration of keratinocytes, we investigated the effect of ATP11B on keratinocytes proliferation. Initially, we confirmed the efficiency of silencing ATP11B (Si ATP11B) by qRT–PCR and Western Blot (WB) (Figure S3A,B). The expression of proliferation marker *Ki–67* was downregulated when the expression of ATP11B was silenced (Figure 4A). Scratch assays also showed that decreased ATP11B expression inhibited cell migration (Figure 4B,C). In addition, the Transwell migration assay also confirmed that silencing ATP11B inhibited keratinocytes migration (Figure 4D). In addition, our results also show that ATP11B is involved in regulating cell cycle progression, and decreased ATP11B expression significantly reduces the G2 phase of cells (Figure 4E). These data suggest that ATP11B is a negative regulator of keratinocytes proliferation and migration. Wound margin tissue mobilize keratinocytes proliferation and migration to promote EMT. Therefore, we further investigated whether miR–618 could impact the process of wound healing by influencing EMT. We analyzed the mRNA expression levels of EMT–related genes. The results from qPCR showed that the overexpression of miR–618 significantly increased the mRNA level of *E–cadherin* and decreased the mRNA levels of *N–cadherin*, *Vimentin*, and *Snail* (Figure 4F). Conversely, the inhibition of miR–618 expression promoted EMT (Figure 4G). In addition, we silenced the expression of ATP11B in keratinocytes. The qPCR results showed that mRNA levels of *N–cadherin*, *Vimentin*, and *Snail* were significantly downregulated in the transfected Si ATP11B group (Figure 4H). Finally, the co–transfection of Si ATP11B and miR–618 inhibitor showed excellent rescue results. Compared with the Si ATP11B group, the protein expression levels of N–cadherin, Vimentin, and Snail were restored, and the expression level of E–cadherin protein was inhibited in co-transfected with Si ATP11B and miR-618 inhibitor (Figure 4I). The inhibition of miR–618 partially reversed the inhibition effect of proliferation and migration in Si ATP11B group. Together, these findings support miR–618 directly targeting ATP11B to inhibit EMT in keratinocytes.

### 2.5. miR–618 Invovles in the PI3K–Akt Signaling Pathway

**Figure 5:**
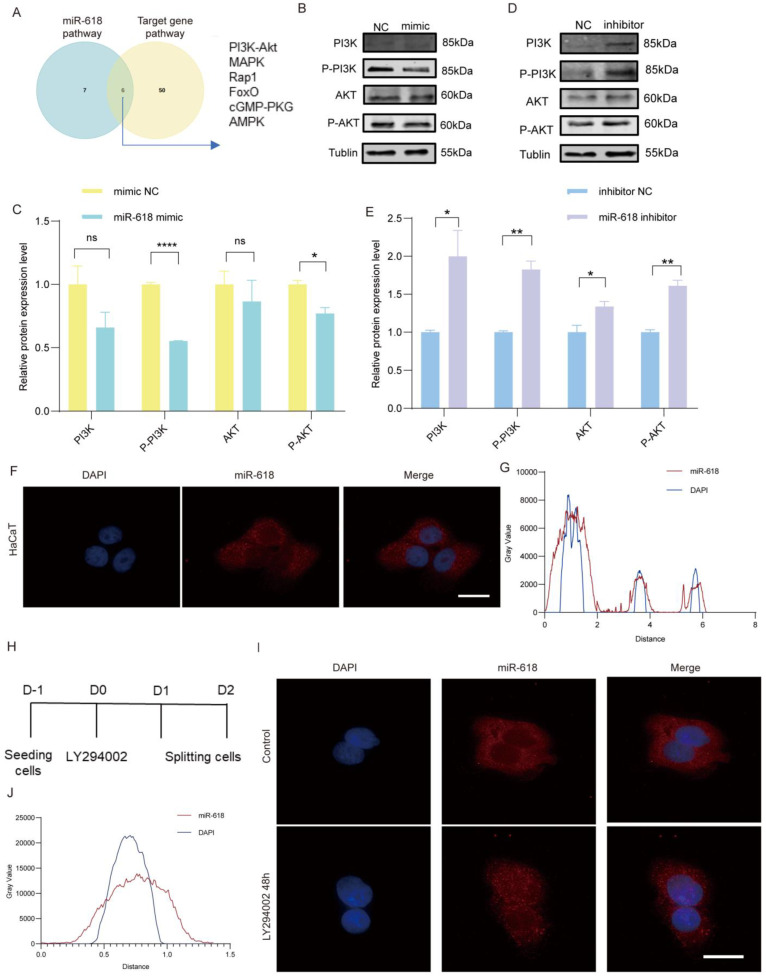
miR−618 invovles in the PI3K−Akt signaling pathway. (**A**) The Venn diagram shows the overlapping signaling pathways enriched by miR−618 and signaling pathways enriched by target genes of miR–618. (**B**,**C**) WB assay was performed to detect the protein expression levels of PI3K, P–PI3K, AKT, and P–AKT in the PI3K–Akt pathway in keratinocytes transfected with miR–618 mimic and NC. Image J 1.53 analyzed the bands’ grayscale values and counted the target proteins’ relative expression levels, using Tublin as the internal reference protein. (**D**,**E**) WB was performed to detect the protein expression levels of PI3K, P–PI3K, AKT, and P–AKT in the PI3K–Akt pathway in keratinocytes transfected with miR–618 inhibitor and NC. Image J 1.53 analyzed the bands’ grayscale values and counted the target proteins’ relative expression levels, using Tublin as the internal reference protein. (**F**) FISH assay to detect the expression level of miR–618 in keratinocytes using FISH probes. Red represents miR–618; blue represents DAPI. Scale: 20 µm. (**G**) Gray FISH values for miR–618 and DAPI in keratinocytes. Red represents miR–618; blue represents DAPI. (**H**) Diagram of LY294002 (inhibitor of PI3K) treating keratinocytes. (**I**) FISH assay to detect the expression level of miR–618 with treated by LY294002. Red represents miR–618; blue represents DAPI. Scale: 20 µm. (**J**) Gray FISH values for miR–618 and DAPI in keratinocytes treated with LY294002. Red represents miR–618; blue represents DAPI. Data are expressed as the mean ± SD. ^ns^ *p* > 0.05, * *p* < 0.05, ** *p* < 0.01, **** *p* < 0.0001. *n* = 3.

To further investigate the signaling pathways involved in the regulation of miR–618, we combined the KEGG analysis of differential miRNA sequencing data from damaged tissues during the inflammatory phase with the KEGG analysis of miR–618 target genes (Figure 5A). Among them, the activation of the PI3K–Akt signaling pathway can promote keratinocytes EMT and promote wound repair. Research shows that the PI3K–Akt signaling pathway has been found to be increasingly important in the triggering transduction cascade affecting miR–618 [11,12,13,14,15]. We hypothesize that miR–618 will target the PI3K–Akt signaling pathway involved in regulating EMT. We examined protein expression levels of P–PI3K, PI3K, P–AKT, and AKT. The WB results showed that P–PI3K and P–AKT were significantly downregulated in the miR–618 overexpression group, while decreasing the expression of miR–618 produced the opposite results (Figure 5B–E). miR–618 can regulate PI3K–Akt signaling pathway–mediated EMT, thereby regulating keratinocytes function. In addition to the widespread expression of miRNAs in the cytoplasm, some mature miRNAs were also found in the nucleus. The FISH experiment confirmed that miR–618 is widely expressed in the nucleus and cytoplasm (Figure 5F,G). To assess whether the subcellular distribution of miR–618 might be affected by the activation of the PI3K–Akt signaling cascade, we measured the miR–618 expression level in the cytoplasm and nucleus of keratinocytes treated with PI3K inhibitor (LY294002) (Figure 5H). Notably, we found that when PI3K–Akt signaling was turned off, and the expression level of miR–618 was significantly enriched in the nucleus (Figure 5I,J). The above data indicate that miR–618 invovled in the PI3K–Akt signaling pathway and plays a role in the nucleus.

### 2.6. miR–618 Binds to the Promoter of Lin7a 

**Figure 6:**
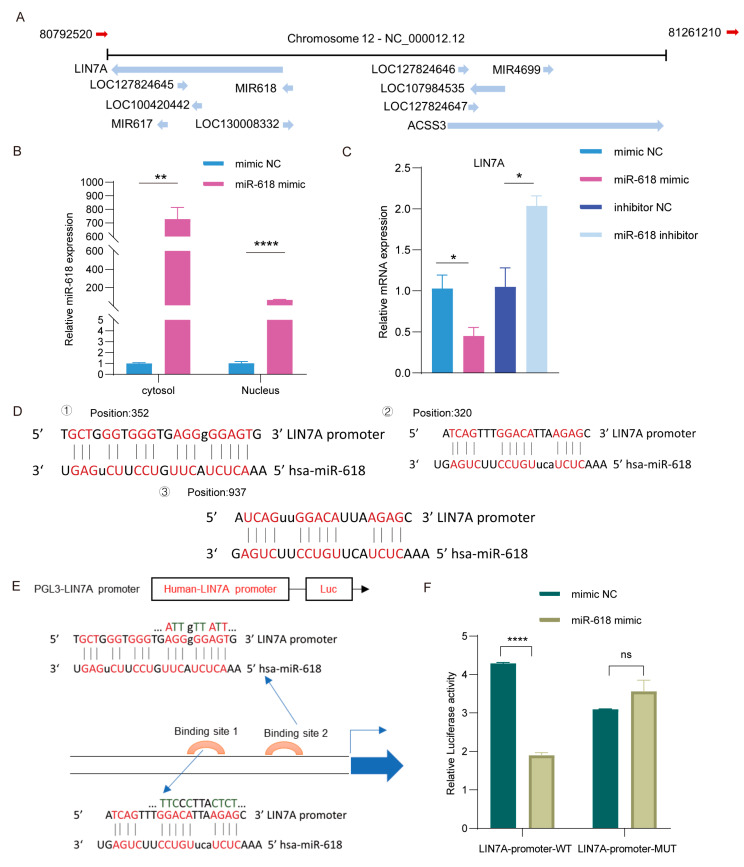
miR–618 binds to the promoter of Lin7a. (**A**) Image of the location of miR–618 chromosome on the UCSC Genome Browser website. (**B**) qPCR assay was used to detect the nuclear and cytoplasmic distribution of miR–618 in keratinocytes transfected with miR–618 mimic and NC. (**C**) qPCR assay was used to detect the mRNA expression level of *Lin7a* in keratinocytes transfected with miR–618 mimic, inhibitor, and NC. (**D**) Sequence alignment of miR–618 binding sites with *Lin7a* promoter. (**E**,**F**) Dual luciferase activity in HEK–293T cells co–transfected with miR–618 mimic and *Lin7a* promoter–WT or *Lin7a* promoter–MUT. Data are expressed as the mean ± SD. ^ns^ *p* > 0.05, * *p* < 0.05, ** *p* < 0.01, **** *p* < 0.0001. *n* = 3.

The regulation of gene transcription is crucial for tissue development and wound repair, including the PI3K–Akt signaling pathway, which has been shown to regulate gene transcription [22]. We screened the gene on the chromosome where miR–618 is located and studied the alteration of its epigenetic state. Interestingly, *Lin7a* is the EMT–related gene closest to miR–618 on the same chromosome–Chr12, which can be found in the UCSC genome browser upstream of miR–618 (Figure 6A). In addition, we found that the expression level of miR–618 in the nucleus was significantly increased after the overexpression of miR–618 (Figure 6B). The mRNA expression of *Lin7a* was significantly downregulated in human keratinocytes treated with miR–618 mimic (Figure 6C). Nuclear miRNAs have been reported to induce transcriptional gene silencing (TGS) at the transcriptional level. To specifically clarify the mechanism of miR–618 in the nucleus and the functional significance of the expression of *Lin7a* regulated by miR–618, the binding site and sequence of miR–618 and the *Lin7a* promoter (mfe < −20 kcal/mol) were predicted through the RNAhybird online website (Figure 6D). Next, we selected two binding site mutations and constructed the pGL3 plasmid carrying the *Lin7a* promoter (Figure 6E). When miR–618 was overexpressed, it increased the luciferase activity of pGL3–Lin7a promoter–WT, but it had no effect on the mutant version (Figure 6F). These results suggest that nuclear miR–618 can regulate the transcription level of *Lin7a* by binding to the *Lin7a* promoter region.

### 2.7. Inhibition of miR–618 Promotes Skin Wound Healing In Vivo

**Figure 7:**
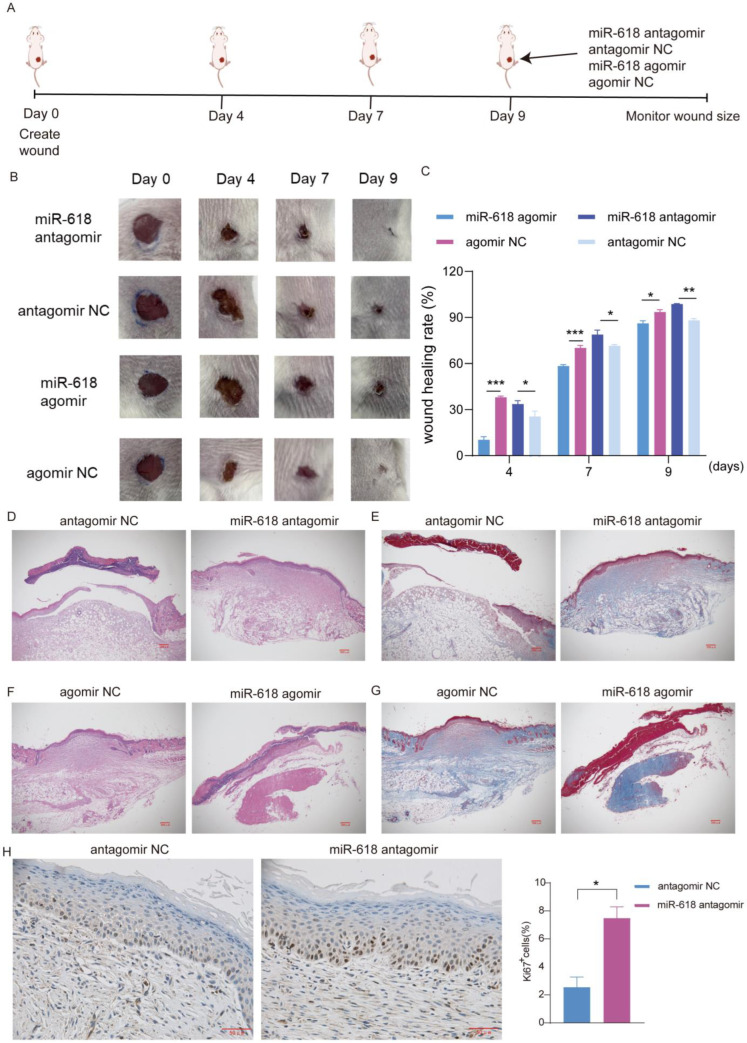
Inhibition of miR–618 promotes skin wound healing in vivo. (**A**) Treatment of wound injury model in mice. miR–618 agomir, antagomir, or NC (50 nM) were injected subcutaneously on day 1 and day 5, respectively (*n* = 3). (**B**) The wound healing of mice in different treatment groups on days 0, 4, 7 and 9 was recorded. (**C**) The statistical situation of the wound healing area of mice, and the healing rate of wound area on day “n” compared with the initial wound area, where n = 4 or 9. (**D**) HE staining analysis showed the wound tissue structure of mice treated with miR–618 antagomir and NC on day 9. Scale: 200 μm. (**E**) Masson staining analysis showed collagen fibers and muscle fibers of mice treated with miR–618 antagomir and NC on day 9. Scale: 200 μm. (**F**) HE staining analysis showed the wound tissue structure of mice treated with miR–618 agomir and NC on day 9. Scale: 200 μm. (**G**) Masson staining analysis showed collagen fibers and muscle fibers of mice treated with miR–618 agomir and NC on day 9. Scale: 200 μm. (**H**) On the left, Ki67 immunohistochemical test showed epidermal cell proliferation in mice treated with miR–618 antagomir and NC on day 9. The right figure shows the Ki67 positive cell rate statistics. Scale: 50 μm. Data are expressed as the mean ± SD. * *p* < 0.05, ** *p* < 0.01, *** *p* < 0.001. *n* = 3.

Finally, the effect of miR–618 expression on wound healing was investigated using an in vivo model. Mice are injected subcutaneously with miR–618 antagomir, agomir, or control oligonucleotides (Figure 7A). We found that wounds treated with miR–618 antagomir showed a significant ability to promote wound closure compared to control–treated wounds, while wounds treated with miR–618 agomir showed a significantly slower wound closure rate compared to control (Figure 7B,C). In addition, the wound tissue of mice on the 9th day of trauma was collected and analyzed by HE and Masson staining. We observed that compared with the control group, miR–618 antagomir–treated mice basically completed wound re–epithelialization and formed a dense collagen fiber network. In contrast, the miR–618 agomir treatment group had thinner skin structure and still had blood scabs (Figure 7D–G). Based on the results above, it can be concluded that reduced miR–618 expression speeds up skin wound healing, while increased miR–618 expression delays the healing process. Furthermore, the immunohistochemical findings revealed a significant increase in the number of Ki67–positive cells when miR–618 expression was inhibited, indicating that inhibiting miR–618 expression promotes the EMT process of epidermal cells (Figure 7H). This study suggests that miR–618 regulates wound healing by affecting the EMT of keratinocytes.

## 3. Discussion

Several studies have shown that miRNA is dynamically changed during wound healing and is specifically involved in the regulation of hemostasis, inflammation, proliferation, and remodeling stages [23,24]. By modulating a single miRNA, it is possible to target multiple targets or a group of functionally relevant genes in a signaling pathway [25], which is highly effective compared to traditional drug treatments. Furthermore, the expression level of miRNA can be effectively inhibited in vitro and in vivo [26]. Therefore, it is necessary to explore more dynamic dysregulation miRNAs that play a further regulatory role in the process of skin injury repair and promote the study of miRNAs as therapeutic agents. In this study, miRNA sequencing data from wound tissue samples at different stages in the GSE database was used to reveal that the expression of miR–618 is dynamically dysregulated in the inflammatory. From the current research reports, miR–618 has important physiological functions and is associated with some tissue cancers and immune diseases [11,12,13,14,15]. Moreover, studies have shown that miR–618 is highly upregulated in plasmacytoid dendritic cells (pDC)in patients with systemic sclerosis, leading to pDC infiltration and skin fibrotic lesions [16]. This suggests that miR–618 is involved in cellular inflammatory response and disrupts tissue homeostasis. We elucidated the inhibition of the wound healing function of miR–618 in vitro and in vivo. In terms of molecular mechanisms, we explored the role of miR–618 in the cytoplasm and nucleus, respectively. In the cytoplasm, we found that miR–618 targeted *Atp11b* to inhibit the PI3K–Akt pathway. In the nucleus, miR–618 binds to the *Lin7a* promoter to regulate the transcription of *Lin7a.*

Current reports suggest that miR–618 is primarily based on studies of tumor cells and organ fibrosis [11,12,13,14,15,16,27]. The role of miR–618 in wound healing has not been reported. Our study found that miR–618 was significantly upregulated during the wound inflammation phase. This is consistent with previous findings that miR–618 is involved in cellular inflammatory responses, leading to pro–inflammatory cytokine storms [16]. *Atp11b* has the highest score in the predicted targeted binding of genes. *Atp11b* has been reported to be closely related to cancer, neurological diseases, cell morphology, and other diseases [20,21]. The “skin–brain” axis reveals the existence of a communication network between the skin and the central nervous system [28], and *Atp11b* has the potential to lead to cognitive and behavioral impairment by delaying skin wound healing. Our results confirm that *Atp11b* can act as a target gene of miR–618 to regulate keratinocytes’ EMT during wound healing. In response to injury pathology, EMT is a key process involved in skin wound healing, activating and mobilizing quiescent keratinocytes in the skin towards the wound site [6]. This is consistent with our results that silencing *Atp11b* inhibits keratinocytes’ proliferation and migration and is a positive regulatory gene for EMT. PI3K–Akt is a classic EMT signaling pathway that mediates EMT in cancer and wound healing [29], and our results also confirm that miR–618 inhibits the PI3K–Akt signaling pathway and leads to keratinocytes’ dysfunction.

While studying the role of miR–618 in the cytoplasm, we made an unexpected discovery—the PI3K–Akt signaling pathway triggers the accumulation of miR–618 in the cell nucleus. This finding aligns with previous research demonstrating that the PI3K–Akt pathway modulates gene accumulation in the nucleus [22]. *Lin7a* is a cancer–associated protein that plays an important role in cell migration by regulating cell polarity [30,31]. This mechanism extends insights into miR–618 regulation during wound healing. Nuclear miR–618 is directly enriched in the promoter region of *Lin7a*, regulates the transcription of *Lin7a* in the nucleus.

However, our mechanistic study is based on in vitro cell models and cannot simulate the real human wound microenvironment. In the future, it may be possible to further increase the complexity of our in vitro models and incorporate multi–omics data, leveraging organoid and organ–on–a–chip technologies, conducting animal model studies, and establishing bridges between in vitro and in vivo research. These may be able to reduce the discrepancies between in vitro findings and in vivo realities. In addition, the specific nuclear epigenetic mechanisms need to be further explored. It is noteworthy that as a key factor in inhibiting wound healing, whether miR–618 has the opportunity to manipulate miRNAs’ expression by delivering miRNA inducers or inhibitor is a future research direction. Therefore, miRNAs have the potential to become the next generation of nucleic acid therapies for wound–healing treatment and management, with the successful development of safe and efficient delivery systems.

## 4. Materials and Methods

### 4.1. Cell Culture

Keratinocytes and HEK–293T cells were cultured in DMEM high glucose (10% FBS, 1% penicillin/streptomycin) at 37 °C at 5% CO_2_.

### 4.2. Plasmid Transfection

ATP11B silencing plasmid vector and its negative control NC, miR–618 mimic, inhibitors, and its NC were purchased from Guangzhou Ruibo Biotechnology Co., Ltd. (Guangzhou, China). miR–618 antagomir, agomir, and its NC were purchased from Shanghai Qingke Biotechnology Co., Ltd (Shanghai, China).

Lipo2000^TM^ reagent was used to transfect the plasmid. The transfection of six–well plate cells was taken as an example and transfected with Lipo2000^TM^ reagent (Beyotime, Shanghai, China). The transfection system for each well was a plasmid (2 μg), miR–618 mimic, inhibitor, and siRNA (20 nM). The plasmid was diluted with opti–MEM medium (50:1) for 5 min at room temperature. Lipo2000^TM^ reagent was gently mixed with plasmid solution for 20 min. The culture medium was changed after transfection for 6 h.

### 4.3. Cell Proliferation and Clone Formation Assays

Cell Counting Kit–8 (Cat No. 40203; Yeasen, Shanghai, China) was used to test cell proliferation according to the manufacturer’s protocol. Transfected cells were collected and seeded on 96–well plates (2 × 10^3^ cells/well). Then, 10 μL of cck8 staining solution was added at different times and incubated at 37 °C for 1.5 h, and OD 450 nm was measured. For the colony formation experiment, cells were seeded at a density of 10^3^ cells/well on six–well plates. After 14 days, cells were fixed with methanol for 15 min, washed with PBS, and stained with 1% crystal violet for 10 min.

### 4.4. Cell Migration and Scratch Assays

The cell migration experiment was performed in the Transwell chamber (Corning Costar, Tewksbury, MA, USA). First, 300 μL of serum–free medium containing 10^4^ cells was seeded in the upper chamber and 500 μL of medium containing 15% FBS in the lower chamber. After incubation at 37 °C for 24 h, the cells were fixed with anhydrous methanol for 10 min; then, the methanol was removed and stained with crystal violet solution for 30 min. For the cell scratch test, the transfected cells were seeded in a six–well plate and scratched with 200 μL pipette tips, keeping the width of the scratches consistent. After scratch processing, the cell migration image was recorded and was analyzed by Image J 1.53.

### 4.5. Western Blotting

The cells were cleaned with cold PBS for three times and were split on ice with RIPA lysate (Beyotime, Shanghai, China) for 30 min. The supernatant was collected by centrifugation (4 °C, 12,000 rpm), and the protein concentration of the samples was determined by a BCA kit (Vazyme Biotech, Nanjing, China). Then, 5 × SDS loading buffer was mixed with the sample (4:1) and boiled at 99 °C for 10 min. The expression levels of ATP11B, E–cadherin, N–cadherin, Snail, Vimentin, PI3K, P–PI3K, AKT, p–AKT, Tublin, and Vinculin were determined by using a PAGE Gel rapid preparation kit (Epizyme Biotech, Shanghai, China). Details of antibodies are shown in Table S1.

### 4.6. Dual Luciferase Reporter Gene Assay

The dual luciferase assay was used to verify the relationship between the *Lin7a* promoter region, *Atp11b,* and miR–618. The binding sequences of the *Lin7a* promoter region and *Atp11b* with miR–618 were predicted by RNA hybridization software, and binding sites were screened by binding free energy. The luciferase reporter gene and miR–618 mimic (20 nM) were transfected into keratinocytes in a 10:1 ratio. The DL101–01 dual luciferase reporter assay kit (Vazyme Biotech, Nanjing, China) was used to assay the reporter gene activity. The cells were lysed by cell lysis buffer, and then, Luciferase Substrate and Renilla Substrate solutions were added sequentially to detect the ratio of fluorescein intensity between firefly fluorescein and sea fluorescein intensity.

### 4.7. Fluorescence In Situ Hybridization (FISH)

The miR–618 FISH probe was designed and synthesized by Servicebio (Wuhan, China). The FISH experiment was performed according to the SweAMI manufacturer’s instructions (Servicebio). Specifically, transfected cells were seeded on a cell crawl and fixed with 4%PFA for 30 min. Protease K (20 μg/mL) digested cells at 40 °C, and they were washed three times with PBS. The hybrid solution was pre–hybridized at 37 °C for 1 h, and the miR–618 probe (500 nM) was added for hybridization at 40 °C overnight. Gradient washing took place with different concentrations of SSC solution (2×, 1×, 0.5×) for 10 min, a branch probe hybridization solution was dropped (60 μL) at 40 °C for 50 min, and then, washing took place with different concentrations of SSC solution successively for 10 min. The signal probe was diluted with the hybrid solution (dilution ratio 1:400) and incubated at 40 °C for 3 h, and then washed with SSC solution of different concentrations for 10 min in turn. Finally, DAPI dye solution was added, and it was incubated away from light for 5 min. The sections were then observed under a laser confocal microscope and the images were collected.

### 4.8. Extraction of Nuclear and Cytoplasmic RNA

The cells were first collected and deposited in a 1.5 mL centrifuge tube and centrifuged at 3000 rpm for 3 min, and then, the supernatant was removed by suction. Then, 300 μL of cytoplasmic lysis buffer was added and suspended, then placed on ice for lysis for 10 min. The cell lysate was centrifuged for 15 min at 12,000 rpm in a centrifuge precooled to 4 °C, and the supernatant became the cytoplasmic component. The supernatant was then transferred to a new centrifuge tube to avoid suction precipitation. It was then washed and precipitated with 500 μL of PBS, followed by centrifugation at 1500× *g* for 5 min, and the supernatant was discarded. Finally, 300 μL of 1 × SDS was added, the liquid was mixed, and the nuclear component was obtained. The expression ratio of target genes in the cytoplasm and nucleus was analyzed by qPCR. *Gapdh* and *U6* were used as internal parameters of the cytoplasm and nucleus.

### 4.9. In Vivo Wound Experiments

The 6–8 week–old BALB/c mice were purchased from Shanghai JieSiJie Laboratory Animal Co., Ltd (Shanghai, China). A full–layer excision wound was created for the mice using tweezers and surgical scissors. The mice were randomly divided into four groups and injected subcutaneously with miR–618 agomir, antagomir, and their NC on day 1 and day 5. To calculate the percentage reduction in wound size compared to the original wound, we used the following formula: Wound closure rate = (wound area at day “0” − wound area at day “n” (n = 4, 7, 9))/wound area at day “0” × 100%.

### 4.10. Cell Cycle Assay

After transfection, the cells were digested with pancreatic enzymes to prepare a single–cell suspension. Cells were collected by centrifuge precipitation (1000× *g*, 5 min) and were washed with cooled PBS. Cells were immobilized overnight with pre–cooled ethanol, and cells were collected by centrifugation (1000× *g*, 5 min). With pre–cooled PBS, they were washed three times to remove residual ethanol. Each sample was added to the dyeing working solution (0.5 mL of dyeing buffer + 10 μL of propyl iodide storage solution + 10 μL of RNaseA solution) and mixed in the dark at 37 °C for 30 min. After incubation, flow cytometry was used for detection.

### 4.11. Data Analysis and Statistics

We obtained normal skin and stomatitis tissue miRNA sequencing data from NCBI (www.ncbi.nlm.nih.gov/geo/, accessed on 10 May 2023) with GSE number 174661. R software identification thresholds were used to identify miRNAs on miRNA sequencing results (|log_2_FC| > 0.38, *p*–value < 0.05). Differential miRNAs were identified via the miEAA (https://ccb-compute2.cs.uni-saarland.de/mieaa2/, accessed on 10 May 2023) The target genes of miR–618 were predicted using miRWalk, Targetscan, and miRDB public algorithm software target genes, and the functional enrichment analysis of target genes was performed with R software. The means between different groups were analyzed using Student’s *t*–test and one–way analysis of variance. Statistical analysis was carried out using GraphPad Prism 8 software, and the data were expressed as the mean ± standard deviation (SD). Experimental differences were considered as significant for *p*–value < 0.05.

## Figures and Tables

**Table 1:** List of significant change in miRNAs in 24 h wound tissue compared with normal skin tissue (top ten).

	Gene ID	log_2_(FoldChange)	Padj
Upregulated	hsa–miR–21–3p	3.321718974	1.32 × 10^−71^
	hsa–miR–223–3p	6.300044118	2.15 × 10^−49^
	hsa–miR–223–5p	5.490959426	3.16 × 10^−32^
	hsa–miR–142–5p	2.971024539	5.19 × 10^−13^
	hsa–miR–4521	2.730764038	3.49 × 10^−11^
	hsa–miR–7–5p	1.762200533	1.13 × 10^−10^
	hsa–miR–155–5p	1.751773261	0.000000123
	hsa–miR–618	3.462254638	0.000000421
	hsa–miR–4772–5p	3.944102665	0.00000252
	hsa–miR–92b–3p	1.184238473	0.00000522
Downregulated	hsa–miR–381–3p	−1.647643487	0.000000000227
	hsa–miR–214–5p	−1.586602599	0.000000003580
	hsa–miR–152–3p	−1.173724769	0.000000123
	hsa–miR–452–5p	−1.359895743	0.000000359
	hsa–miR–1298–5p	−2.162631617	0.00000163
	hsa–miR–136–5p	−2.069115903	0.00000522
	hsa–miR–148a–5p	−1.76727337	0.00000675
	hsa–miR–141–3p	−1.171100221	0.0000453
	hsa–miR–203a–5p	−1.735612631	0.0000575
	hsa–miR–708–3p	−1.750610383	0.000112738

## Data Availability

All data needed to evaluate the conclusions in the paper are presented in the paper and/or the Supplementary Materials. The data that support the findings of this study are available from the corresponding author upon reasonable request.

## Supplementary Materials

The following supporting information can be downloaded at https://www.mdpi.com/article/10.3390/ijms25147617/s1.
