# Supplementary material for: Inhibiting miR–618 Promotes Keratinocytes Proliferation and Migration to Enhance Wound Healing in Mice
Source: Int J Mol Sci. 2024 Jul 11;25(14):7617. doi: 10.3390/ijms25147617 (PMC11277496; doi:10.3390/ijms25147617)
Supplement: Supplementary file 1 [file ijms-25-07617-s001.zip › ijms-3044483-supplementary.pdf]

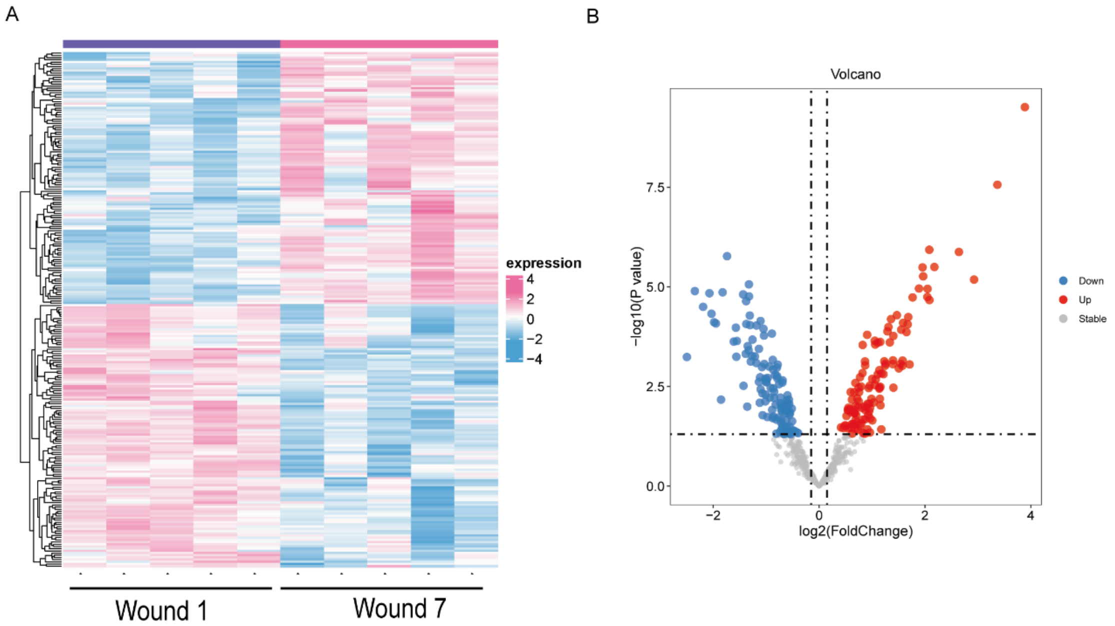

Figure S1 Mapping of miRNA expression in the proliferation phase of human skin wound. (A) Heatmap illustrating of significantly changed miRNAs. Each row represents a miRNA and each column represents a sample, with red repre senting up-regulated miRNA and blue representing down-regulated miRNA. (B) Volcanic map of miRNA expression genes between normal skin tissue and wound tissue in proliferation phase. Red shows up-regulated genes, blue dow n-regulated genes. The x-coordinate represents the difference multiple, and th e y-coordinate represents the p-value of the differential gene. (n = 5).

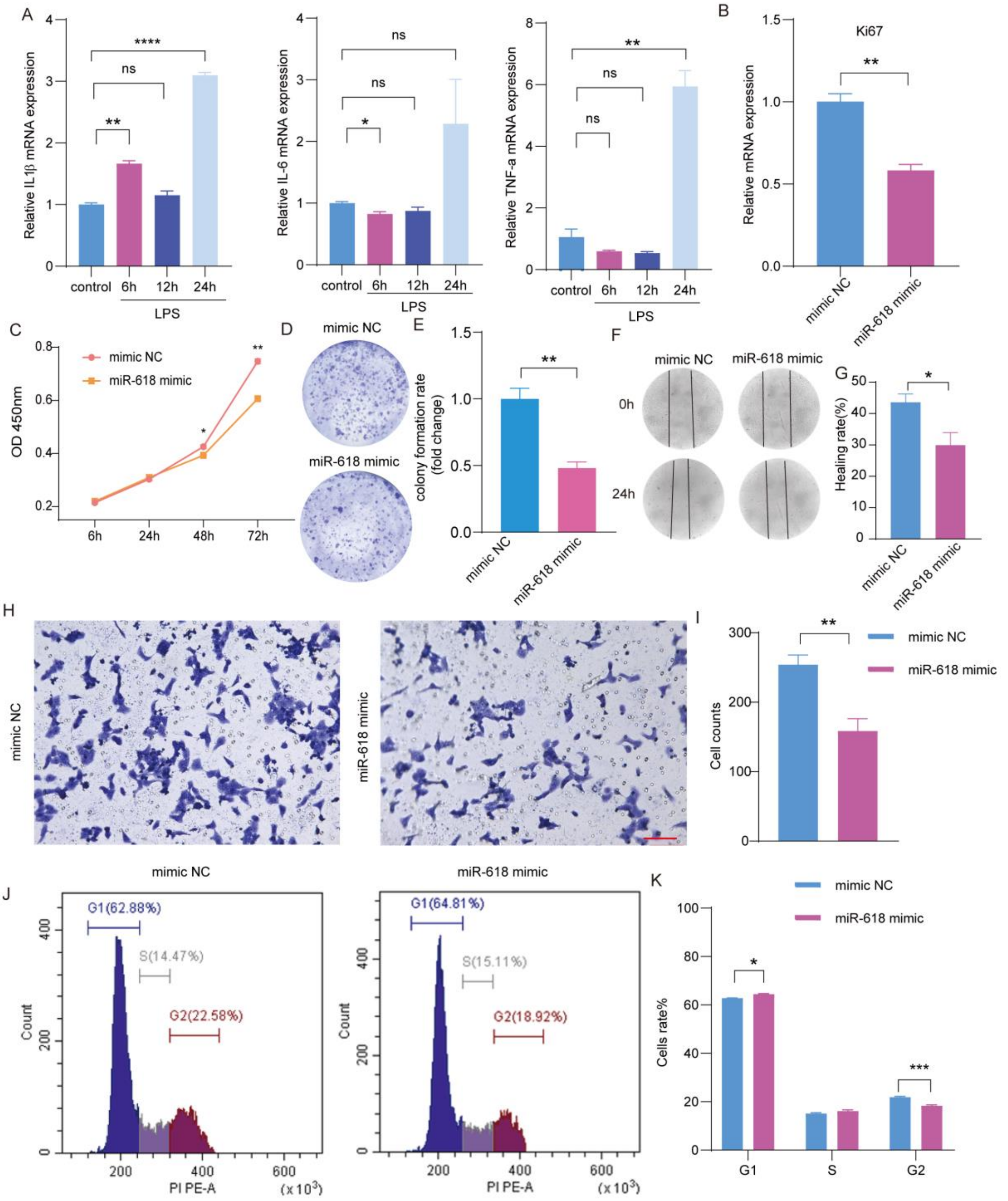

Figure S2 miR-618 regulates the inflammatory and proliferative phases of human skin wound. (A) qPCR assay was used to detect the mRNA expression levels of inflammatory factors after LPS treatment at different times, including *IL-1β*, *IL-6* and *TNF-a*. (B) qPCR assay was used to detect the mRNA expression levels of *Ki67* in keratinocyte cell transfected with miR-618 mimic and NC. (C) CCK-8 assay was performed to evaluate the proliferation ability of keratinocyte cell transfected with miR-618 mimic and NC. (D-E) Colony formation was performed to evaluate the proliferation ability of keratinocyte cell transfected with miR-618 mimic and NC. (F-G) Scratch assay was performed to evaluate the migration ability of keratinocyte transfected with miR-618 mimic and NC. Images were taken at 0 h and 24 h after scratching. (H-I) Transwell assay was performed to evaluate the migration ability of keratinocyte transfected with miR-618 mimic and NC. scale 100 μm. (J-K) The cell cycle of transfected cells was analy

zed by flow cytometry. The percentage of transfected cells in G1, S, and G2 phases of the cell cycle was displayed. Data are expressed as the mean ± SD. ^{ns} *p> 0.05*, *p< 0.05, **p< 0.01, ***p< 0.001, ****p<0.0001. *n*=3.

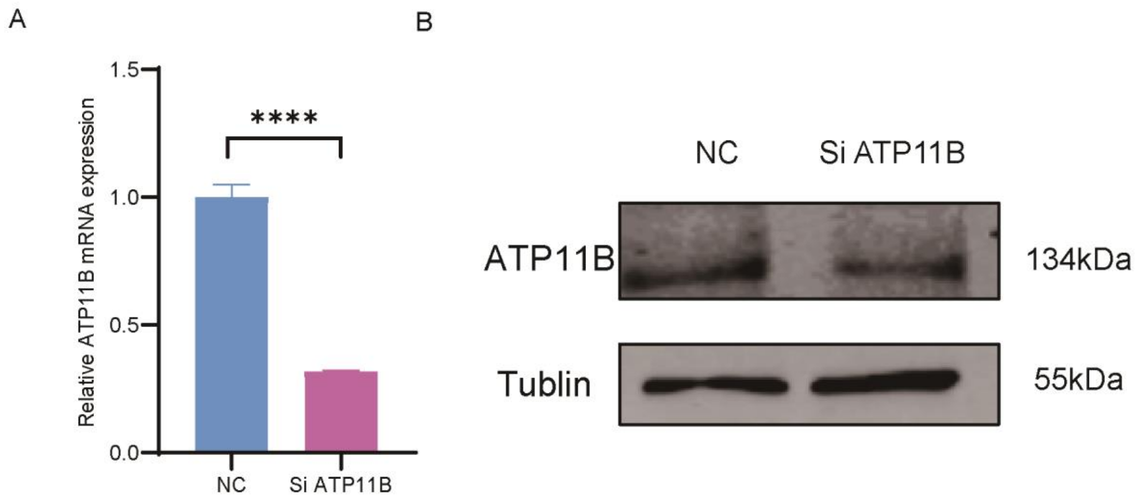

FigureS3 Validation of the silencing effect of *Atp11b* in keratinocyte cell. (A) The mRNA expression level of *Atp11b* was measured by qPCR after transfection of Si ATP11B and its corresponding NC in keratinocyte cell. (B) WB was performed to detect the protein expression level of ATP11B after transfection of Si ATP11B and its corresponding NC in keratinocyte cell. Data are expressed as the mean ± SD. ****p<0.0001. *n*=3.

Table S1: List of Antibodies used in this study

Antibody	Company
Anti-Atp11b	Signalway Antibody
Anti-Vinculin	Signalway Antibody
Anti-β-Tublin	Abways
Anti-E-cadherin	ABclonal
Anti-N-cadherin	ABclonal
Anti-Vimentin	ABclonal
Anti-Snail	ABclonal
Anti-Ki67	Servicebio
Anti-PI3K	Proteintech
Anti-P-PI3K	Abways
Anti-AKT	Abways
Anti-P-AKT	Cell signaling Technology
DAPI	Beyotime

The antibody dilution ratio is diluted according to the instructions.
